# Five-year Outcomes of Magnetic Resonance Imaging–based Active Surveillance for Prostate Cancer: A Large Cohort Study^☆^

**DOI:** 10.1016/j.eururo.2020.03.035

**Published:** 2020-09

**Authors:** Vasilis Stavrinides, Francesco Giganti, Bruce Trock, Shonit Punwani, Clare Allen, Alex Kirkham, Alex Freeman, Aiman Haider, Rhys Ball, Neil McCartan, Hayley Whitaker, Clement Orczyk, Mark Emberton, Caroline M. Moore

**Affiliations:** a Division of Surgery and Interventional Science, University College London, London, UK; b Department of Urology, UCLH NHS Foundation Trust, London, UK; c Department of Radiology, UCLH NHS Foundation Trust, London, UK; d Division of Epidemiology, James Buchanan Brady Urological Institute, Johns Hopkins University, Baltimore, MD, USA; e Department of Histopathology, UCLH NHS Foundation Trust, London, UK

**Keywords:** Prostate cancer, Magnetic resonance imaging, Active surveillance

## Abstract

**Background:**

Although the use of multiparametric magnetic resonance imaging (mpMRI) in active surveillance (AS) for prostate cancer is of increasing interest, existing data are derived from small cohorts.

**Objective:**

We describe clinical, histological, and radiological outcomes from an established AS programme, where protocol-based biopsies were omitted in favour of MRI-led monitoring.

**Design, setting, and participants:**

Data on 672 men enrolled in AS between August 2004 and November 2017 (inclusion criteria: Gleason 3 + 3 or 3 + 4 localised prostate cancer, presenting prostate-specific antigen <20 ng/ml, and baseline mpMRI) were collected from the University College London Hospital (UCLH) database.

**Outcome measurements and statistical analysis:**

Primary outcomes were event-free survival (EFS; event defined as prostate cancer treatment, transition to watchful waiting, or death) and treatment-free survival (TFS). Secondary outcomes included rates of all-cause or prostate cancer–related mortality, metastasis, and upgrading to Gleason ≥4 + 3. Data on radiological and histological progression were also collected.

**Results and limitations:**

More than 3800 person-years (py) of follow-up were accrued (median: 58 mo; interquartile range 37–82 mo). Approximately 84.7% (95% confidence interval [CI]: 82.0–87.6) and 71.8% (95% CI: 68.2–75.6) of patients remained on AS at 3 and 5 yr, respectively. EFS and TFS were lower in those with MRI-visible (Likert 4–5) disease or secondary Gleason pattern 4 at baseline (log-rank test; *p* <  0.001). In total, 216 men were treated. There were 24 deaths, none of which was prostate cancer related (6.3/1000 py; 95% CI: 4.1–9.5). Metastases developed in eight men (2.1 events/1000 py; 95% CI: 1.0–4.3), whereas 27 men upgraded to Gleason ≥4 + 3 on follow-up biopsy (7.7 events/1000 py; 95% CI: 5.2–11.3).

**Conclusions:**

The rates of discontinuation, mortality, and metastasis in MRI-led surveillance are comparable with those of standard AS. MRI-visible disease and/or secondary Gleason grade 4 at baseline are associated with a greater likelihood of moving to active treatment at 5 yr. Further research will concentrate on optimising imaging intervals according to baseline risk.

**Patient summary:**

In this report, we looked at the outcomes of magnetic resonance imaging (MRI)-based surveillance for prostate cancer in a UK cohort. We found that this strategy could allow routine biopsies to be avoided. Secondary Gleason pattern 4 and MRI visibility are associated with increased rates of treatment. We conclude that MRI-based surveillance should be considered for the monitoring of small prostate tumours.

## Take Home Message

We report excellent clinical outcomes from one of the largest imaging-based active surveillance cohorts to date. Magnetic resonance imaging (MRI)-visible cancer at baseline, along with 3 + 4 Gleason grade, predicts a greater likelihood of treatment at five years. MRI-visible lesions appear to have a distinct clinical trajectory from less conspicuous ones.

## 1 Introduction

Active surveillance (AS) is an established approach for managing prostate cancers of low-intermediate risk in men who want to defer surgery or radiotherapy. Mature AS cohorts indicate a low risk of prostate cancer mortality or adverse outcomes of delayed radical treatment [1], [2], [3], [4]. However, approximately 40% of men have treatment within 5 yr, primarily due to disease progression [5]. In addition, underestimation of baseline risk with standard biopsy and the morbidity associated with serial tissue sampling remain significant challenges in AS.

Multiparametric magnetic resonance imaging (mpMRI) is diagnostically superior to standard transrectal ultrasonography-guided (TRUS) biopsy [6], [7]. In the AS setting, mpMRI (1) improves baseline risk allocation by identifying men who need early treatment [8], (2) detects progression in men on surveillance [9], and (3) reduces the need for serial biopsies [10]. MRI is being introduced in several AS protocols [11]. The UK National Institute for Care and Clinical Excellence (NICE) now recommends mpMRI at baseline for all AS candidates and for the assessment of clinical or prostate-specific antigen (PSA) changes during surveillance [12]. Incorporation of mpMRI appears to be cost effective and may be beneficial in overcoming the anxiety that some men experience in the early AS years [13], [14].

Existing imaging-based cohorts are limited by their small size and relatively short follow-up [9]. We describe clinical, radiological, and histological results from a large population where the central strategy was not to perform protocol-based biopsies, but to base follow-up on PSA and MRI, with further sampling only in cases of radiological change or unexplained PSA fluctuations.

## 2 Patients and methods

### 2.1 Participants

The University College London Hospitals (UCLH) AS programme includes men with (1) Gleason 3 + 3 or Gleason 3 + 4 prostate cancer, (2) PSA < 20 ng/ml, and (3) baseline mpMRI (the date of which marks entry into imaging-based AS). Histological diagnosis of cancer is achieved through standard TRUS biopsies, transperineal biopsies, and, occasionally, transurethral resections (transurethral resection of the prostate) followed by further confirmatory sampling. MRI findings are reported using an ordinal Likert score expressing the likelihood of clinically significant cancer (1, “highly unlikely”; 2, “unlikely”; 3, “indeterminate” or “equivocal”; 4, “likely”; and 5, “highly likely”). Suspicious MRI lesions must be concordant with biopsy findings before starting AS (ideally using an MRI-targeted approach), whilst men without lesions are not routinely offered a biopsy.

**Fig. 1:**
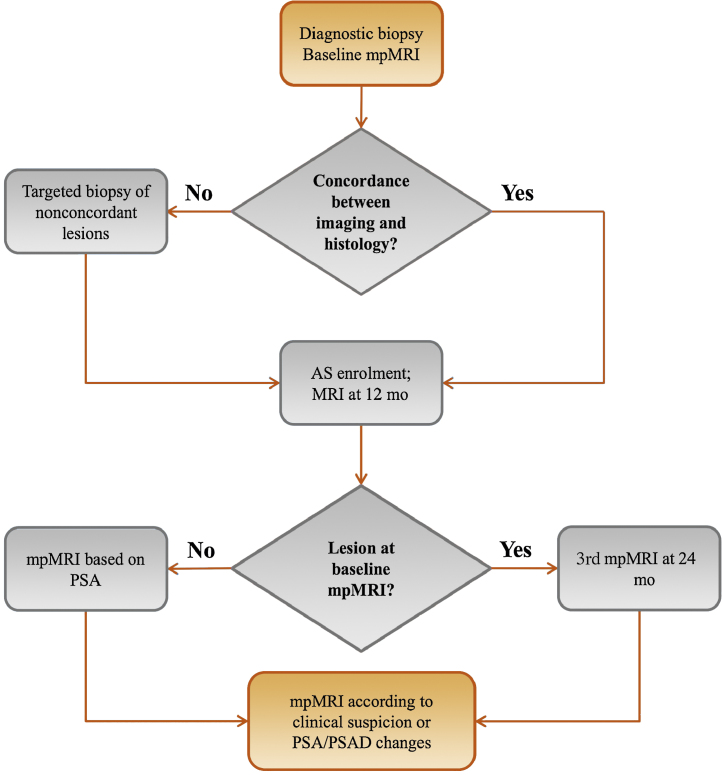
UCLH AS protocol overview. All men are offered baseline mpMRI (the date of which marks entry into imaging-based AS) and second MRI at 12 mo in accordance with NICE guidelines. The decision to perform additional MRI scans is informed by baseline imaging and clinical/PSA changes. AS = active surveillance; mpMRI = multiparametric MRI; MRI = magnetic resonance imaging; NICE = National Institute for Care and Clinical Excellence; PSA = prostate-specific antigen; PSAD = PSA density; UCLH = University College London Hospital.

The history, MRI scans, and biopsy outcomes of AS candidates are reviewed at a multidisciplinary meeting comprising urologists, uroradiologists, and uropathologists. Once eligibility is confirmed, patients are monitored through mpMRI and PSA according to a standard protocol that takes into account baseline visibility: MRI is performed at baseline and at 12 mo, and then, depending on the presence of visible disease, at 24 mo, with PSA-based MRI later (Fig. 1). Additional biopsies are carried out in cases of concerning radiological, clinical, or PSA changes, and are performed using a transperineal, MRI-targeted approach. If histological progression (ie, upgrade to Gleason ≥4 + 3 and/or increase in maximum cancer core length) is confirmed, patients are routinely offered treatment, ranging from traditional interventions (radical prostatectomy, radiotherapy, and hormones) to focal treatments (high-intensity focal ultrasound and cryotherapy) and on-trial experimental modalities (photodynamic therapy, radiofrequency ablation, and injectable compound administration). This work is part of the continuous service evaluation programme within our unit.

### 2.2 Study design

The records of 672 consecutive men who attended UCLH for AS between August 2004 and November 2017 were considered. Data on clinical parameters, PSA measurements, clinic letters and information on any prostate cancer treatment, transition to watchful waiting (WW), metastasis, or death until April 31, 2019, were collected. Data were supplemented with information from other hospitals or general practitioners where necessary.

The primary outcomes were event-free survival (EFS; “event” defined as any prostate cancer treatment, transition to WW, Gleason ≥4 + 3 on follow-up biopsy, or any death) and treatment-free survival. Censoring was defined as the date of the last recorded clinical appointment. Secondary outcomes included rates of all-cause or prostate cancer–related death (defined as any death reported in records or clinical letters as either definitely or possibly relevant to prostate cancer or its treatment), metastasis (defined as nodal or bone foci on nuclear imaging), transition to WW, and Gleason ≥4 + 3 on follow-up biopsy.

Histology data were extracted from pathology reports, including biopsy method, Gleason grade, positive/total cores, and maximum cancer core length. Similarly, data were extracted from radiological reports, including prostate volume, presence of “MRI-visible” disease (defined as reference to a well-defined lesion or Likert score 4–5), and radiological progression (defined as any reference to “progressing appearances”, increased overall Likert score, new MRI-visible areas, increasing lesion size, or increasingly restricted diffusion of an existing lesion). Outcomes were stratified by baseline Gleason grade (3 + 3 vs 3 + 4) and disease MRI visibility (“nonvisible disease” vs “visible disease”).

### 2.3 Statistical analysis

The specific study questions of interest were to describe clinical, radiological, and histological outcomes in the cohort, as well as to estimate EFS and treatment-free survival. Summary statistics (proportions, medians, and interquartile ranges [IQRs]) were derived where relevant, and confidence intervals (CIs) for proportions were estimated with continuity correction at the 95% level. The Kaplan-Meier method was used to estimate the proportion of individuals remaining on AS at different time points, whereas differences between strata (Gleason grade and MRI visibility) were tested through the log-rank procedure. All *p* values were significant at the 0.05 level. Analyses were performed in Microsoft Excel 2010 (Microsoft Corporation, Redmond, WA, USA) and R (R Foundation for Statistical Computing, Vienna, Austria; URL: https://www.R-project.org/).

## 3 Results

### 3.1 Cohort characteristics

In total, 524 men (78%) had Gleason 3 + 3 at baseline, whilst the remaining 148 (22%) had Gleason 3 + 4 cancer (Table 1). Diagnosis was made through standard TRUS in 453/672 men (67.4%). Men with Gleason 3 + 4 were older, had higher PSA/PSA density, and a greater proportion had MRI-visible disease at baseline (56.1%), compared with the Gleason 3 + 3 group (40.5%). Of note, 384 out of 672 men (57.1%) had received a diagnosis and undergone a short period of surveillance (median: 7 mo; IQR 4–18 mo) before their first mpMRI. Median follow-up for the cohort as a whole was 58 mo (IQR 37–82 mo), whereas for men without the primary event of interest, median follow-up was 63 mo (IQR 44–88 mo).

**Table 1:** Cohort baseline characteristics (*n* = 672).^a^

	Gleason 3 + 3		Gleason 3 + 4	
*n*	524		148	
Likert 4–5 at 1 st mpMRI, *n* (%)	212	(40.5)	83	(56.1)
Age at diagnosis (yr), *n* (range)	62	(56–66)	64	(58.75–70.25)
Presenting PSA (ng/ml), *n* (range)	6	(4.5–8·4)	6.9	(5.17–8.9)
Baseline PSAD (ng/ml^2^), *n* (range)	0.12	(0.09–0.18)	0.14	(0.1–0.22)
Tissue diagnosis, *n* (%)				
TRUS	363	(69.3)	90	(60.8)
TPM	118	(22.5)	36	(24.3)
TP	23	(4.4)	13	(8.8)
TURP/other	20	(3.8)	9	(6.1)
Targeted	101	(19.3)	24	(16.2)
Positive cores	2	(1–3)	3	(2–5)
Total cores	12	(10–22)	12	(12–32)
MCCL	1.5	(1–3)	4	(2–6)

IQR = interquartile range; MCCL = maximum cancer core length; mpMRI = multiparametric magnetic resonance imaging; PSA = prostate-specific antigen; PSAD = PSA density; TP = transperineal; TPM = template prostate mapping; TRUS = transrectal ultrasonography; TURP = transurethral resection of the prostate.

a Baseline characteristics of men included in the study (*n* = 672). Absolute numbers or medians are given for age, PSA, PSAD, number of positive/total cores, and MCCL, with IQRs or percentages in parentheses. The majority of patients had Gleason 3 + 3 cancer and, of those, 40.5% had MRI-visible disease at baseline (defined as a Likert score 4–5 or a clearly outlined lesion as described by the reporting radiologist). Men with Gleason 3 + 4 cancer were older and had higher PSA/PSAD, and a greater proportion had visible disease at baseline compared with the Gleason 3  + 3 group.

### 3.2 Summary of clinical events

An initial overview of clinical outcomes is presented in Table 2. In total, 216 men (32.1%) underwent treatment (62.0 patients/1000 person-years; 95% CI: 54.3–70.6). Ninety-four men had radical treatment (62 radical prostatectomy, 27 external beam radiotherapy—with or without androgen deprivation, and five seed brachytherapy). Furthermore, 106 men had focal therapy (86 high-intensity focused ultrasound, 12 cryotherapy, and eight other on-trial modalities). Sixteen men had androgen deprivation therapy alone.

**Table 2:** Clinical outcomes for the entire AS cohort, stratified according to Gleason grade and MRI visibility at baseline.^a^

	Gleason grade at baseline	All patients	Nonvisible disease		MRI-visible disease	
			3 + 3	3 + 4	3 + 3	3 + 4
*n*		672	312	65	212	83
						
Mortality	Prostate cancer–related deaths	0	0	0	0	0
	Deaths due to other causes	24	8	4	5	7
	Total person-year follow-up	3811				
	All-cause deaths/1000 py (95% CI)	6.3 (4.1–9.5)				
						
Metastasis	Nodal	5	1	0	2	2
	Bone	3	0	1	1	1
	Total person-year follow-up	3792				
	Total metastatic events/1000 py (95% CI)	2.1 (1.0–4.3)				
						
Treatment	Number of patients treated	216	68	17	82	49
	Total person-year follow-up	3486				
	Patients treated/1000 py (95% CI)	62.0 (54.3–70.6)				
						
	RP	62	21	4	23	14
	EBRT (±ADT)	27	8	1	12	6
	ADT alone	16	5	0	6	5
	Brachytherapy	5	1	1	3	0
	Focal	106	33	11	38	24
						
Transition to WW		21	5	3	9	4
						
Upgrade to Gleason ≥4 + 3	Upgrading on follow-up biopsy	27	11	2	7	7
	Total person-year follow-up	3514				
	Upgrading on follow-up biopsies/1000 py (95% CI)	7.7 (5.2–11.3)				

ADT = androgen deprivation therapy; AS = active surveillance; CI = confidence interval; EBRT = external beam radiotherapy; IQR = interquartile range; MRI = magnetic resonance imaging; py = person-years; RP = radical prostatectomy; WW = watchful waiting.

a Metastasis and prostate cancer–related death were overall rare events, but their rates were higher in the Gleason 3 + 4 and/or “MRI-visible” disease groups. Treatment rates were higher in patients with an MRI lesion at baseline. Most treated patients underwent focal therapy, predominantly high-intensity focused ultrasound. Out of 21 men who transitioned to WW, only one was previously upgraded to Gleason ≥4 + 3, whereas the rest transitioned to WW due to comorbidities, advanced age, or personal preference. IQRs, percentages, or 95% confidence intervals (with continuity correction) are given in parentheses.

**Fig. 2:**
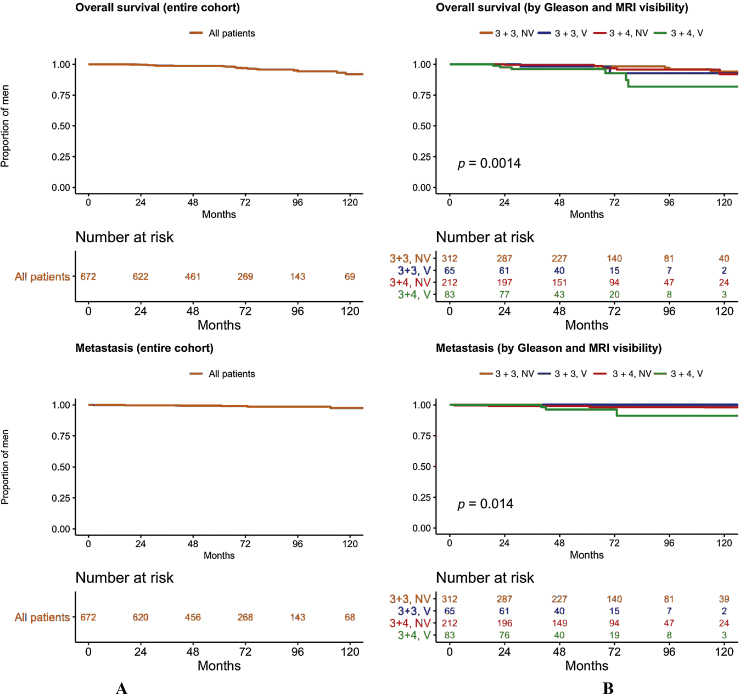
Overall survival and time to metastasis (A) for the entire cohort and (B) stratified by Gleason and baseline MRI findings. Metastasis was defined as any nodal or bone metastatic focus on a bone or PET scan. Overall survival and metastasis-free survival were high for the cohort as a whole, but there was a significant difference between the four groups (3 + 3 N V, 3 + 3 V, 3 + 4 N V, and 3 + 4 V; log-rank test, *p* <  0·05). This was primarily driven by lower survival in the Gleason 3 + 4 group; there was no difference between men with and without a visible lesion at baseline in either grade group (separate log-rank analyses, not shown). MRI = magnetic resonance imaging; NV = non-visible disease; PET = positron emission tomography; V = visible disease.

There were no prostate cancer–related deaths. There were 24 deaths from other causes, primarily cardiovascular disease or other malignancies (6.3 deaths/1000 person-years; 95% CI: 4.1–9.5). The Gleason 3 + 4/MRI-visible group had a higher rate of all-cause death (Fig. 2A and 2B). There were eight metastatic events (2.1 events/1000 person-years; 95% CI: 1.0–4.3), and metastasis was more common in the Gleason 3 + 4/MRI-visible group (Fig. 2C and 2D). Twenty-seven men were upgraded to Gleason ≥4 + 3 (7.7 events/1000 person-years, 95% CI: 5.2–11.3). Twenty-six of these men underwent treatment, whereas one transitioned to WW due to comorbidities. In total, 21 men transitioned to WW (seven due to personal preference, one due to advanced age, and the others because of comorbidities).

### 3.3 Primary outcomes: EFS and treatment-free survival

**Fig. 3:**
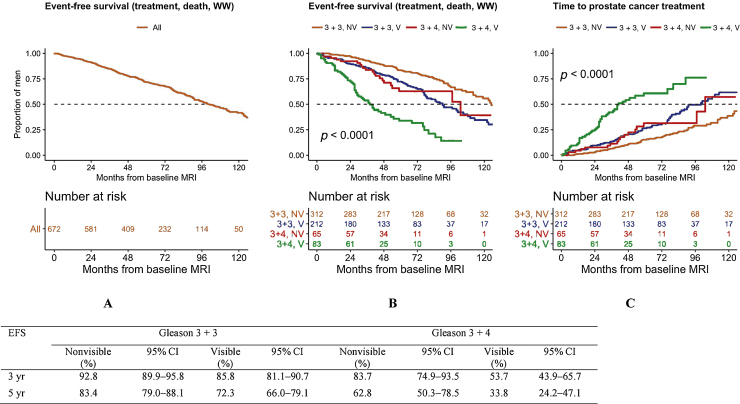
**Figure and Table 3: Event-free survival (EFS):** Kaplan-Meier curves of time to treatment, transition to WW or death for the entire cohort (left) and stratified by baseline Gleason grade and MRI visibility (middle) are shown. There was a significant difference in EFS between the 4 groups (log-rank test, *p* < 0.001) and although men with Gleason 3+4 cancer had a different trajectory to those with Gleason 3+3, MRI-visible disease at baseline was associated with shorter EFS in both Gleason groups. EFS estimates at three and five years with 95% CI are given in the table. **Treatment-free survival:** Cumulative curves for any prostate cancer treatment stratified by baseline Gleason and MRI visibility are shown (right). When treating WW and death as competing risks, Gleason and MRI visibility were significant predictors of time to treatment whereas age and PSAD were not; age, however, was the strongest predictor of death or transition to WW (separate analyses, not shown). CI = confidence interval; EFS = event-free survival; HR = hazard ratio; MRI = magnetic resonance imaging; PSAD = prostate-specific antigen density; WW = watchful waiting.

Approximately 84.7% (95% CI: 82.0–87.6) and 71.8% (95% CI: 68.2–75.6) of all patients remained on AS at 3 and 5 yr, respectively (Fig. 3). EFS was significantly lower in the MRI-visible and/or Gleason 3 + 4 groups (log rank test, *p* <  0.001): in men with Gleason 3 + 3 cancer, the 5-yr EFS was 83.4% and 72.3% for those with nonvisible and visible disease, respectively, whereas in the Gleason 3 + 4 group, these rates were 62.8% and 33.8%, respectively (Fig. 3). In the vast majority of cases, treatment was prompted by radiological and pathological progression, followed by treatment on the basis of MRI change alone in selected men.

### 3.4 Histological outcomes and radiological change

**Fig. 4:**
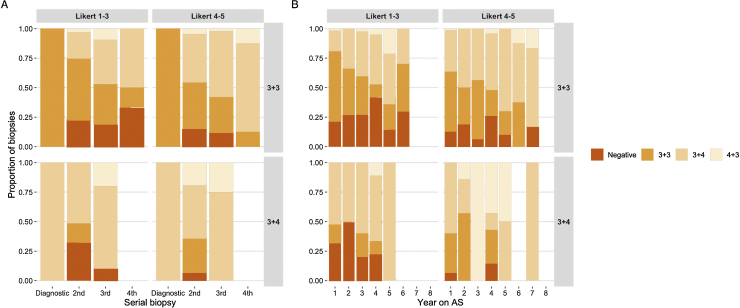
Histological outcomes in MRI-based AS, stratified by baseline Gleason grade and disease visibility on MRI. Proportions of biopsies with a diagnosis of no cancer, Gleason 3 + 3, Gleason 3 + 4, and Gleason ≥4 + 3 cancer are shown. The absolute number of biopsies performed declined over time as patients discontinued AS (Supplementary Tables 2A and 2B). (A) Serial biopsy data: Gleason 3 + 4 and ≥4 + 3 disease were both proportionately more prevalent in follow-up biopsies of men initially diagnosed with MRI-visible disease and/or Gleason 3 + 4 cancer. Gleason 3 + 4 patients did not have a third serial biopsy. (B) Yearly biopsy data (AS years 1–8). Of note, a few biopsies (including some with upgrading) were performed beyond year 8. Gleason upgrading was consistently more frequent in men initially diagnosed with MRI-visible disease, particularly if they belonged to the Gleason 3 + 4 group. Upgrading events in the latter group generally occurred earlier (years 3–5) than in those with initial Gleason 3 + 3 (years 4–7). On the whole, negative biopsies were more frequent in the “non-visible” (Likert 1–3) groups. AS = active surveillance; MRI = magnetic resonance imaging.

**Fig. 5:**
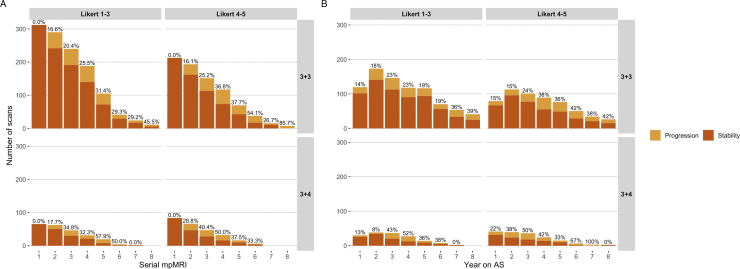
Radiological change in MRI-based AS, stratified by baseline Gleason grade and disease visibility on mpMRI. The total number of MRI scans performed decreases over time as more men leave the cohort. **(A)** The proportion of MRI scans reported as demonstrating radiological progression (rounded percentages, top of each bar) increased with each additional MRI scan, particularly in men diagnosed with Gleason 3 + 4 and a visible MRI lesion at baseline. **(B)** Similar increasing trends were noted for each additional year on imaging-based AS. In both Gleason groups, MRI progression was a more frequent event in men with visible disease at baseline. A substantial number of MRI scans were performed between 0 and 24 mo (NICE guidelines advocate second MRI approximately 12 mo after the first). AS = active surveillance; mpMRI = multiparametric MRI; MRI = magnetic resonance imaging; NICE = National Institute for Care and Clinical Excellence.

The outcomes of all biopsies and MRI scans were derived in both serial and yearly format, and stratified according to baseline Gleason and MRI visibility (Supplementary Tables 1 and 2). The majority of diagnostic biopsies were TRUS guided, but there was a transition to more extensive sampling (such as template mapping) at the first follow-up biopsy and a gradual shift towards targeted approaches over time. The proportion of Gleason 3 + 4 or ≥4 + 3 diagnoses increased with each additional biopsy and for each additional year on AS (Fig. 4). However, upgrading events predominantly occurred in the Gleason 3 + 4/MRI-visible group and clustered in earlier AS years. In parallel, the proportion of MRI scans reported as “radiologically progressing” increased with each additional MRI scan and each additional year on AS (Fig. 5). These proportions were consistently higher in the Gleason 3 + 4/MRI-visible group and clustered in earlier AS years. There was a gradual increase in PSA and prostate volume in all groups, but there was no obvious longitudinal PSA density pattern.

## 4 Discussion

We reported medium-term outcomes in a large mpMRI-based AS cohort with more than 3800 person-years of follow-up. This cohort is unique due to its inclusion criteria, protocol, size, and risk stratification according to baseline imaging. Prostate cancer death and metastasis at five years were low, as might be expected [1], [2], [3], [4].

We showed that most patients, particularly those with Gleason 3 + 3 cancer and nonvisible disease at baseline, remained on imaging-based surveillance at five years, whereas the leading cause of AS discontinuation was combined radiological and pathological progression. Only one patient in our cohort underwent treatment due to anxiety, corroborating the clinical experience at our institution where men tend to be reassured by serial mpMRI scans. The treatment rate was similar to that reported from standard AS cohorts with comparable follow-up [3], [4]. An assessment of over 10 000 men on AS estimated the cumulative incidence of progression to be 27.5% and a dropout rate of 43.6% at 5 yr, which is higher than in our study [5]. In other AS studies, 5-yr conversion rates are even higher [15]. By comparison, small imaging-based studies have reported active treatment rates of 27.5% (with median time to treatment 4.2 yr) and 30% (with median time to treatment 1.55 yr) [16], [17]. Some authors report treatment rates as low as 11.7%, with the majority of men treated at a yearly rate of <4% after the first 2 yr (with a median follow-up of 39 mo) [18]. We showed that Gleason grade and MRI visibility at baseline are associated with EFS and treatment-free survival, a finding previously confirmed by others [17]. We also believe that WW after AS should not necessarily be viewed as a “failure”, but often as a “graduation” event.

The more stringent monitoring of MRI-visible disease and a higher likelihood of a biopsy in these men could introduce an ascertainment bias in our study and could drive the higher rates of upgrading or treatment in the “MRI-visible” groups. However, it is known that MRI visibility of disease in AS candidates confers a higher probability of adverse pathology at surgery, which indicates an association between MRI phenotypes and biological features [19]. We also found that radiological progression was more frequent in the “MRI-visible” groups. Therefore, it could be more likely for pre-existing lesions to worsen rather than for new lesions to emerge, a finding supported by other authors [20]. Similarly, our observation that upgrading is more frequent in men with positive baseline MRI has been described previously by others [17].

A limitation of our study was heterogeneity in the methods used for tissue diagnosis. However, all biopsy and MRI results of AS candidates are regularly reviewed, and, in cases of discordance, repeat tests ensure safe risk stratification and a high degree of consistency. The Likert scale was used instead of the popular Prostate Imaging Reporting and Data System (PIRADS) version 2 reporting tool. However, there is significant experience with Likert in our institution and this system was used consistently from the beginning of the study until its end, which could also explain why the year of diagnosis was not a significant predictor of EFS despite major changes in technology and reporting practices that occurred in the past decade (separate analyses, not shown). The latest NICE guidance recommends Likert for MRI reporting, and comparisons with PIRADS have shown that both systems have similar inter-reader variability and reproducibility [21], [22]. Whilst Likert 3 is often used to denote positive MRI in the decision for an initial biopsy, we deemed Likert 4 or 5 more appropriate in this AS population, where a major aim is to detect Gleason ≥4 + 3 disease [23]. We used a loose definition of radiological progression based on radiological reports and not on stringent criteria such as the Prostate Cancer Radiological Estimation of Change in Sequential Evaluation (PRECISE) system [24].

The single-centre, retrospective nature of this study, along with the relatively high between-centre variability in mpMRI interpretation, limits the generalisability of our findings, and we do not advocate the uncritical adoption of our protocol in every clinical setting. Nonetheless, we provide an overview of MRI-led AS outcomes in a cohort monitored according to NICE guidelines and supplied evidence that baseline imaging characteristics could have predictive value. We acknowledge that longitudinal trends in AS patients determine clinical management and could influence the time to clinical outcome. However, the introduction of time-dependent covariates or joint longitudinal-survival models was beyond the scope of this work and requires many assumptions to be met, including knowledge of data missingness mechanisms. There are, however, excellent efforts to apply such methods in AS [25].

## 5 Conclusions

Approximately 85% and 72% of patients remain on MRI-led AS at 3 and 5 yr, respectively. MRI visibility and Gleason grade are determining factors of EFS and time to prostate cancer treatment. MRI-visible cancer lesions appear to have a distinct radiological, pathological, and clinical trajectory, but further research is required in order to determine true differences in the natural history of MRI-visible and MRI-invisible prostate cancer.

  ***Author contributions*:** Vasilis Stavrinides had full access to all the data in the study and takes responsibility for the integrity of the data and the accuracy of the data analysis.

*Study concept and design*: Stavrinides, Moore, Giganti.

*Acquisition of data*: Stavrinides, Giganti, Kirkham, Punwani, Allen, Freeman, Ball, Haider.

Analysis and interpretation of data: Stavrinides, Moore, Giganti.

Drafting of the manuscript: Stavrinides, Moore.

*Critical revision of the manuscript for important intellectual content*: Trock, Giganti, Kirkham, Punwani, Allen, Freeman, Ball, Haider, Orczyk, Emberton.

*Statistical analysis*: Stavrinides, Trock.

*Obtaining funding*: Stavrinides, Moore.

Administrative, technical, or material support: McCartan.

*Supervision*: Moore, Whitaker, Orczyk, Emberton.

*Other*: None.

  ***Financial disclosures:*** Vasilis Stavrinides certifies that all conflicts of interest, including specific financial interests and relationships and affiliations relevant to the subject matter or materials discussed in the manuscript (eg, employment/affiliation, grants or funding, consultancies, honoraria, stock ownership or options, expert testimony, royalties, or patents filed, received, or pending), are the following: None.

  ***Funding/Support and role of the sponsor*:** Vasilis Stavrinides is supported by an MRC Clinical Research Training Fellowship (MR/S005897/1) and has previously been supported by a UCL Bogue Fellowship and an EACR Travel Fellowship. Francesco Giganti is funded by the UCL Graduate Research Scholarship and the Brahm PhD scholarship in memory of Chris Adams. Alex Kirkham is supported by the UCLH/UCL Biomedical Research Centre. Mark Emberton is a United Kingdom National Institute of Health Research (NIHR) Senior Investigator and receives research support from the UCLH/UCL NIHR Biomedical Research Centre. Caroline M. Moore is supported by the UK NIHR, Movember, PCUK, and the EAU Research Foundation.

## CRediT authorship contribution statement

**Vasilis Stavrinides:** Conceptualization, Methodology, Software, Formal analysis, Investigation, Data curation, Writing - original draft, Writing - review & editing, Visualization. **Francesco Giganti:** Validation, Investigation, Data curation, Writing - review & editing. **Bruce Trock:** Methodology, Formal analysis. **Shonit Punwani:** Investigation, Resources. **Clare Allen:** Investigation, Resources. **Alex Kirkham:** Investigation, Resources. **Alex Freeman:** Investigation, Resources. **Aiman Haider:** Investigation, Resources. **Rhys Ball:** Investigation, Resources. **Neil McCartan:** Investigation, Resources. **Hayley Whitaker:** Supervision, Writing - review & editing, Funding acquisition. **Clement Orczyk:** Supervision, Writing - review & editing. **Mark Emberton:** Supervision, Writing - review & editing, Funding acquisition, Project administration. **Caroline M. Moore:** Resources, Supervision, Writing - review & editing, Funding acquisition, Project administration.
