# Supplementary material for: Five-year Outcomes of Magnetic Resonance Imaging–based Active Surveillance for Prostate Cancer: A Large Cohort Study[image]
Source: Eur Urol. 2020 Sep;78(3):443–51. doi: 10.1016/j.eururo.2020.03.035 (PMC7443696; doi:10.1016/j.eururo.2020.03.035)
Supplement: Supplementary file 1 [file mmc1.docx]

| Supplementary Table 1A: Serial biopsy outcomes stratified by Gleason grade and disease MRI visibility at baseline. | | | | | | | | | | | |
| --- | --- | --- | --- | --- | --- | --- | --- | --- | --- | --- | --- |
| Gleason | **Visible** | **Biopsy** |  | **Diagnostic** | | **1st follow up** | | **2nd follow up** | | **3rd follow up** | |
| 3+3 | **No** | **Months from baseline** |  | - |  | 13 | (3-36.75) | 40 | (20.75-64) | 76 | (69-92) |
|  |  | **Number of biopsies** |  | 312 |  | 164 |  | 53 |  | 6 |  |
|  |  | **Approach** | **TRUS** | 219 | (70.2%) | 21 | (12.8%) | 4 | (7.5%) | 1 | (16.7%) |
|  |  |  | **TPM** | 71 | (22.8%) | 83 | (50.6%) | 13 | (24.5%) | - | - |
|  |  |  | **TP** | 8 | (2.6%) | 53 | (32.3%) | 34 | (64.2%) | 4 | (66.7%) |
|  |  |  | **TURP/Other** | 14 | (4.5%) | 7 | (4.3%) | 2 | (3.8%) | 1 | (16.7%) |
|  |  |  | **Targeted** | 43 | (13.8%) | 94 | (57.3%) | 41 | (77.4%) | 4 | (66.7%) |
|  |  | **Outcome** | **3+3** | 312 | (100%) | 86 | (52.4%) | 18 | (34%) | 1 | (16.7%) |
|  |  |  | **3+4** | - | - | 37 | (22.6%) | 20 | (37.7%) | 3 | (50%) |
|  |  |  | **≥ 4+3** | - | - | 5 | (3.0%) | 5 | (9.4%) | - | - |
|  |  |  | **+ve cores** | 1.0 | (1-3) | 2.5 | (1-6) | 3 | (1-7.25) | 4.5 | (3.75-7) |
|  |  |  | **Total cores** | 12 | (10-22.5) | 17 | (11-47) | 12 | (8.25-24.25) | 11.5 | (10.25-13.5) |
|  |  |  | **MCCL** | 1 | (1-2) | 2 | (1-5) | 3 | (2-6) | 5 | (4.25-5.5) |
|  | **Yes** | **Months from baseline** |  | - |  | 10 | (3-40) | 37.5 | (29.25-54) | 71 | (63.25-73.25) |
|  |  | **Number of biopsies** |  | 212 |  | 127 |  | 43 |  | 8 |  |
|  |  | **Approach** | **TRUS** | 144 | (67.9%) | 19 | (15%) | 1 | (2.3%) | - | - |
|  |  |  | **TPM** | 47 | (22.2%) | 55 | (43.3%) | 13 | (30.2%) | - | - |
|  |  |  | **TP** | 15 | (7.1%) | 51 | (40.2%) | 26 | (60.5%) | 8 | (100%) |
|  |  |  | **TURP/Other** | 6 | (2.8%) | 2 | (1.6%) | 3 | (7%) | - | - |
|  |  |  | **Targeted** | 58 | (27.4%) | 94 | (74%) | 37 | (86%) | 8 | (100%) |
|  |  | **Outcome** | **3+3** | 212 | (100%) | 50 | (39.4%) | 13 | (30.2%) | 1 | (12.5%) |
|  |  |  | **3+4** | - | - | 52 | (40.9%) | 24 | (55.8%) | 6 | (75%) |
|  |  |  | **≥ 4+3** | - | - | 6 | (4.7%) | 1 | (2.3%) | 1 | (12.5%) |
|  |  |  | **+ve cores** | 2 | (1-3) | 4 | (2-6) | 4 | (3-9) | 5.5 | (3.75-7.75) |
|  |  |  | **Total cores** | 12 | (11-22) | 17 | (10-44) | 14 | (8-28) | 11 | (9-12.25) |
|  |  |  | **MCCL** | 2 | (1-4) | 4 | (2-6) | 6 | (4-8) | 6.5 | (4.25-8.25) |
| 3+4 | **No** | **Months from baseline** |  | - |  | 8 | (4-22) | 46.5 | (37.25-49.5) | - |  |
|  |  | **Number of biopsies** |  | 65 |  | 31 |  | 10 |  | - |  |
|  |  | **Approach** | **TRUS** | 42 | (64.6%) | 2 | (6.5%) | - | - | - |  |
|  |  |  | **TPM** | 16 | (24.6%) | 20 | (64.5%) | 2 | (20%) | - |  |
|  |  |  | **TP** | 2 | (3.1%) | 8 | (25.8%) | 7 | (70%) | - |  |
|  |  |  | **TURP/Other** | 5 | (7.7%) | 1 | (3.2%) | 1 | (10%) | - |  |
|  |  |  | **Targeted** | 4 | (6.2%) | 19 | (61.3%) | 9 | (90%) | - |  |
|  |  | **Outcome** | **3+3** | - | - | 5 | (16.1%) | - | - | - |  |
|  |  |  | **3+4** | 65 | (100%) | 16 | (51.6%) | 7 | (70%) | - |  |
|  |  |  | **≥ 4+3** | - | - | - | - | 2 | (20%) | - |  |
|  |  |  | **+ve cores** | 2 | (1-4) | 3 | (1-6) | 4 | (2-9) | - |  |
|  |  |  | **Total cores** | 14 | (12-39.5) | 26 | (12-48) | 12 | (8-20) | - |  |
|  |  |  | **MCCL** | 3 | (2-4) | 3 | (2-4) | 5 | (2-7) | - |  |
|  | **Yes** | **Months from baseline** |  | - |  | 13.5 | (2.25-30.5) | 40.5 | (32.5-51.5) | - |  |
|  |  | **Number of biopsies** |  | 83 |  | 31 |  | 4 |  | - |  |
|  |  | **Approach** | **TRUS** | 48 | (57.8%) | 2 | (6.5%) | - | - | - |  |
|  |  |  | **TPM** | 20 | (24.1%) | 8 | (25.8%) | 1 | (25%) | - |  |
|  |  |  | **TP** | 11 | (13.3%) | 21 | (67.7%) | 3 | (75%) | - |  |
|  |  |  | **TURP/Other** | 4 | (4.8%) | - | - | - | - | - |  |
|  |  |  | **Targeted** | 20 | (24.1%) | 26 | (83.9%) | 3 | (75%) | - |  |
|  |  | **Outcome** | **3+3** | - | - | 9 | (29.0%) | - | - | - |  |
|  |  |  | **3+4** | 83 | (100%) | 14 | (45.2%) | 3 | (75%) | - |  |
|  |  |  | **≥ 4+3** | - | - | 6 | (19.4%) | 1 | (25%) | - |  |
|  |  |  | **+ve cores** | 4 | (2-6) | 5.5 | (2.75-8) | 4.5 | (4-5.5) | - |  |
|  |  |  | **Total cores** | 12 | (10.25-32) | 13 | (10-25) | 14 | (9.25-21) | - |  |
|  |  |  | **MCCL** | 4 | (3-6) | 6.25 | (3-9.25) | 4.5 | (3.75-5.5) | - |  |

| **Supplementary Table 1B: Yearly biopsy outcomes (first 8 years), stratified by Gleason grade and disease MRI visibility at baseline.** | | | | | | | | | | | | | | | | | | |
| --- | --- | --- | --- | --- | --- | --- | --- | --- | --- | --- | --- | --- | --- | --- | --- | --- | --- | --- |
| **Gleason** | **Visible** | **Months from baseline** | | **0-12** |  | **13-24** |  | **25-36** |  | **37-48** |  | **49-60** |  | **61-72** |  | **73-84** |  | **85-96** |
| **3+3** | **No** | **Biopsies** |  | 104 |  | 56 |  | 36 |  | 18 |  | 14 |  | 10 |  | - |  | - |
|  |  | **Approach** | **TRUS** | 15 | (14.4%) | 6 | (10.7%) | 2 | (5.6%) | 0 | - | 1 | (7.1%) | 1 | (10%) | - |  | - |
|  |  |  | **TPM** | 61 | (58.7%) | 13 | (23.2%) | 4 | (11.1%) | 3 | (16.7%) | 5 | (35.7%) | 0 | - | - |  | - |
|  |  |  | **TP** | 23 | (22.1%) | 34 | (60.7%) | 28 | (77.8%) | 13 | (72.2%) | 8 | (57.1%) | 8 | (80%) | - |  | - |
|  |  |  | **TURP/Other** | 5 | (4.8%) | 3 | (5.3%) | 2 | (5.6%) | 2 | (11.1%) | 0 | - | 1 | (10%) | - |  | - |
|  |  |  | **Targeted** | 54 | (51.9%) | 40 | (71.4%) | 30 | (83.3%) | 16 | (88.9%) | 12 | (85.7%) | 8 | (80%) | - |  | - |
|  |  | **Outcome** | **Negative** | 22 | (21.2%) | 15 | (27.3%) | 10 | (25%) | 7 | (38.9%) | 2 | (14.3%) | 3 | (30%) | - |  | - |
|  |  |  | **3+3** | 62 | (59.6%) | 22 | (40.0%) | 12 | (33.3%) | 2 | (11.1%) | 3 | (21.4%) | 4 | (40%) | - |  | - |
|  |  |  | **3+4** | 18 | (17.3%) | 19 | (34.5%) | 14 | (38.9%) | 8 | (44.4%) | 6 | (42.9%) | 3 | (30%) | - |  | - |
|  |  |  | **≥ 4+3** | 2 | (1.9%) | 0 | - | 1 | (2.8%) | 1 | (5.6%) | 3 | (21.4%) | 0 | - | - |  | - |
|  |  |  | **+ve cores** | 3 | (1-5) | 3 | (2-8) | 3 | (1-5) | 3 | (1-4) | 3 | (2-9) | 3 | (1.25-5.5) | - |  | - |
|  |  |  | **Total cores** | 39 | (11-52.75) | 11.5 | (7.75-17) | 12 | (7.5-15) | 12 | (7-16) | 17.5 | (11.5-32.25) | 9.5 | (7.75-13.5) | - |  | - |
|  |  |  | **MCCL** | 2 | (1-3.1) | 3 | (2-7) | 4 | (2-7) | 5 | (4-8) | 6 | (2-7) | 3.5 | (2-6.5) | - |  | - |
|  | **Yes** | **Biopsies** |  | 71 |  | 16 |  | 16 |  | 23 |  | 10 |  | 8 |  | 12 |  | - |
|  |  | **Approach** | **TRUS** | 4 | (5.63%) | 2 | (12.5%) | 0 | - | 4 | (17.4%) | 0 | - | 1 | (12.5%) | 0 | - | - |
|  |  |  | **TPM** | 45 | (63.4%) | 8 | (50.0%) | 6 | (40%) | 3 | (13.0%) | 2 | (20%) | 0 |  | 1 | (8.3%) | - |
|  |  |  | **TP** | 21 | (29.6%) | 6 | (37.5%) | 9 | (60%) | 14 | (60.9%) | 8 | (80%) | 7 | (87.5%) | 10 | (83.3%) | - |
|  |  |  | **TURP/Other** | 1 | (1.4%) | 0 | - | 1 | (6.7%) | 2 | (8.7%) | 0 | - | 0 | - | 1 | (8.3%) | - |
|  |  |  | **Targeted** | 40 | (67.8%) | 13 | (81.3%) | 12 | (80%) | 18 | (78.3%) | 10 | (100%) | 8 | (100%) | 11 | (91.7%) | - |
|  |  | **Outcome** | **Negative** | 9 | (12.7%) | 3 | (18.8%) | 1 | (6.7%) | 6 | (26.1%) | 1 | (10%) | 0 | - | 2 | (16.7%) | - |
|  |  |  | **3+3** | 36 | (50.7%) | 5 | (31.3%) | 8 | (53.3%) | 5 | (21.7%) | 2 | (20%) | 3 | (37.5%) | 0 | - | - |
|  |  |  | **3+4** | 25 | (35.2%) | 8 | (50.0%) | 7 | (46.7%) | 11 | (47.8%) | 7 | (70%) | 4 | (50%) | 8 | (66.7%) | - |
|  |  |  | **≥ 4+3** | 1 | (1.4%) | 0 | - | 0 | - | 1 | (4.3%) | 0 | - | 1 | (12.5%) | 2 | (16.7%) | - |
|  |  |  | **+ve cores** | 4 | (2-6.5) | 4 | (2-7) | 4.5 | (3-9.5) | 4 | (3-8) | 5 | (3-7) | 3 | (3-5.5) | 6 | (4.5-7) | - |
|  |  |  | **Total cores** | 44 | (12-58) | 16 | (13.5-53) | 20 | (12-31) | 11.5 | (8.5-17) | 12 | (9-22) | 10 | (6-11.5) | 11 | (8.5-16) | - |
|  |  |  | **MCCL** | 3 | (2-4) | 4 | (2-8) | 6 | (4-9.5) | 7 | (5.5-9) | 8 | (5-9) | 5.5 | (3.5-6.25) | 8 | (5.5-10) | - |
| **3+4** | **No** | **Biopsies** |  | 19 |  | 4 |  | 5 |  | 9 |  | 1 |  | - |  | - |  | - |
|  |  | **Approach** | **TRUS** | 0 | - | 0 | - | 1 | (20%) | 0 | - | 0 | - | - |  | - |  | - |
|  |  |  | **TPM** | 16 | (84.2%) | 3 | (75%) | 2 | (40%) | 2 | (22.2%) | 0 | - | - |  | - |  | - |
|  |  |  | **TP** | 3 | (15.8%) | 1 | (25%) | 2 | (40%) | 5 | (55.6%) | 1 | (100%) | - |  | - |  | - |
|  |  |  | **TURP/Other** | 0 | - | 0 | - | 0 | - | 2 | (22.2%) | 0 | - | - |  | - |  | - |
|  |  |  | **Targeted** | 12 | (66.7%) | 2 | (50%) | 4 | (80%) | 6 | (66.7%) | 1 | (100%) | - |  | - |  | - |
|  |  | **Outcome** | **Negative** | 6 | (31.6%) | 2 | (50%) | 1 | (20%) | 2 | (22.2%) | 0 | - | - |  | - |  | - |
|  |  |  | **3+3** | 3 | (15.8%) | 0 | - | 1 | (20%) | 1 | (11.1%) | 0 | - | - |  | - |  | - |
|  |  |  | **3+4** | 10 | (52.6%) | 2 | (50%) | 3 | (60%) | 5 | (55.6%) | 1 | (100%) | - |  | - |  | - |
|  |  |  | **≥ 4+3** | 0 | - | 0 | - | 0 | - | 1 | (11.1%) | 0 | - | - |  | - |  | - |
|  |  |  | **+ve cores** | 3 | (2-8) | 1 | (0-2.25) | 5 | (1-6) | 3 | (1.5-5.5) | 19 |  | - |  | - |  | - |
|  |  |  | **Total cores** | 42 | (26-53) | 19.5 | (12.5-25.75) | 12 | (11-38) | 15 | (9.5-18) | 29 |  | - |  | - |  | - |
|  |  |  | **MCCL** | 2.5 | (1.4-4) | 2.5 | (2.25-2.75) | 4.5 | (2-7.75) | 4 | (2-4.5) | 8 |  | - |  | - |  | - |
|  | **Yes** | **Biopsies** |  | 15 |  | 7 |  | 2 |  | 7 |  | 2 |  | - |  | 1 |  | - |
|  |  | **Approach** | **TRUS** | 0 | - | 1 | (14.3%) | 0 | - | 0 | - | 0 | - | - |  | 0 | - | - |
|  |  |  | **TPM** | 6 | (40%) | 0 | - | 2 | (100%) | 1 | (14.3%) | 0 | - | - |  | 0 | - | - |
|  |  |  | **TP** | 9 | (60%) | 6 | (85.7%) | 0 | - | 6 | (85.7%) | 2 | (100%) | - |  | 1 | (100%) | - |
|  |  |  | **TURP/Other** | 0 | - | 0 | - | 0 | - | 0 | - | 0 | - | - |  | 0 | - | - |
|  |  |  | **Targeted** | 10 | (66.7%) | 7 | (100%) | 1 | (50%) | 6 | (85.7%) | 2 | (100%) | - |  | 1 | (100%) | - |
|  |  | **Outcome** | **Negative** | 1 | (6.6%) | 0 | - | 0 | - | 1 | (14.3%) | 0 | - | - |  | 0 | - | - |
|  |  |  | **3+3** | 5 | (33.3%) | 4 | (57.1%) | 0 | - | 2 | (28.6%) | 0 | - | - |  | 0 | - | - |
|  |  |  | **3+4** | 9 | (60%) | 2 | (28.6%) | 0 | - | 1 | (14.3%) | 1 | (50%) | - |  | 1 | (100%) | - |
|  |  |  | **≥ 4+3** | 0 | - | 1 | (14.3%) | 2 | (100%) | 3 | (42.9%) | 1 | (50%) | - |  | 0 | - | - |
|  |  |  | **+ve cores** | 7 | (2.5-8) | 4 | (3.5-6.5) | 19 | (18-20) | 5 | (2.5-6) | 6.5 | (5.25-7.75) | - |  | 4 |  | - |
|  |  |  | **Total cores** | 14 | (10-53.5) | 10 | (10-12) | 45.5 | (37.75-53.25) | 17 | (10.5-19) | 9 | (8.5-9.5) | - |  | 7 |  | - |
|  |  |  | **MCCL** | 3 | (2-6.75) | 5 | (3.5-5.5) | 11.5 | (11.25-11.75) | 7.5 | (4.75-9.5) | 8.5 | (7.75-9.25) | - |  | 3 |  | - |

**Supplementary Table 1: Serial (A) and yearly (B; years 1-8) biopsy outcomes of MRI-based AS.** The majority of patients were diagnosed on standard TRUS-guided biopsy, but many subsequently underwent more extensive sampling (including template mapping). There was also a gradual shift towards targeted approaches over time. The number of positive cores and/or the maximum cancer core length (MCCL) increased with each additional biopsy or additional year on surveillance. Some biopsies (including some with Gleason upgrading to ≥ 4+3) were performed beyond year 8. Absolute numbers or medians are shown, with percentages or IQRs (respectively) in brackets.

| **Supplementary Table 2A: Serial mpMRI outcomes stratified by Gleason grade and disease MRI visibility at baseline.** | | | | | | | | | | | | | | | | | | | | |
| --- | --- | --- | --- | --- | --- | --- | --- | --- | --- | --- | --- | --- | --- | --- | --- | --- | --- | --- | --- | --- |
| **Gleason** | **Visible** | **MRI** | **1 (baseline)** | | **2^nd^** | | **3^rd^** | | **4^th^** | | **5^th^** | | **6^th^** | | **7^th^** | | | **8^th^** | | |
| **3+3** | **No** | **Months** | - |  | 14 | (11-22) | 34 | (25-45) | 53 | (41-66) | 69 | (55.25-94) | 86 | (71.5-105.5) | 94 | (87.25-106.5) | 108 | | (94-112.5) |  |
|  |  | **N of scans** | 312 |  | 290 |  | 240 |  | 188 |  | 105 |  | 41 |  | 24 |  | 11 | |  |  |
|  |  | **Lesion** | - |  | 62 | (21.4%) | 52 | (21.7%) | 49 | (26.1%) | 28 | (26.7%) | 14 | (34.1%) | 9 | (37.5%) | 4 | | (36.4%) |  |
|  |  | **Progression** | - |  | 48 | (16.6%) | 49 | (20.4%) | 48 | (25.5%) | 33 | (31.4%) | 12 | (29.3%) | 7 | (29.2%) | 5 | | (45.5%) |  |
|  |  | **PSA** | 5.9 | (4.0-8.3) | 6 | (4.23-8.56) | 7.1 | (4.44-10.32) | 8.0 | (5.0-10.97) | 9.9 | (6.5-14) | 8.5 | (4.91-11) | 11 | (8.85-14.5) | 12.8 | | (9.39-13.3) |  |
|  |  | **Volume** | 47 | (34-66.5) | 52 | (38-72) | 56.5 | (40-82) | 58 | (42-82.5) | 62 | (45-86) | 60.5 | (44-78.25) | 55 | (48-84) | 62 | | (45-76) |  |
|  |  | **PSAD** | 0.11 | (0.08-0.17) | 0.11 | (0.07-0.17) | 0.12 | (0.09-0.18) | 0.12 | (0.09-0.18) | 0.13 | (0.09-0.21) | 0.13 | (0.11-0.19) | 0.18 | (0.13-0.21) | 0.15 | | (0.11-0.17) |  |
|  | **Yes** | **Months** | - |  | 14 | (11-24) | 32 | (24-43) | 50 | (38-61.75) | 62 | (51-78) | 71 | (61-86) | 83 | (77.5-91.5) | 91 | | (82-97) |  |
|  |  | **N of scans** | 212 |  | 193 |  | 151 |  | 117 |  | 69 |  | 37 |  | 15 |  | 7 | |  |  |
|  |  | **Lesion** | 212 |  | 127 | (65.8%) | 80 | (53%) | 63 | (53.8%) | 45 | (65.2%) | 24 | (64.9%) | 9 | (60%) | 5 | | (71.4%) |  |
|  |  | **Progression** | - |  | 31 | (16.1%) | 38 | (25.2%) | 43 | (36.8%) | 26 | (37.7%) | 20 | (54.1%) | 4 | (26.7%) | 6 | | (85.7%) |  |
|  |  | **PSA** | 6.3 | (5-8.97) | 6.94 | (5-10) | 6.7 | (4.95-9.31) | 7.6 | (5.58-10.7) | 9 | (6.2-12) | 9.04 | (5.64-12.88) | 9.85 | (7.99-13) | 11.5 | | (6-18.62) |  |
|  |  | **Volume** | 50 | (37-66.5) | 55 | (37-69) | 57.5 | (42.25-77) | 59 | (44-73) | 68 | (51.75-83.5) | 69 | (44.75-85.5) | 63 | (59-101) | 78.5 | | (72.25-87) |  |
|  |  | **PSAD** | 0.13 | (0.09-0.18) | 0.14 | (0.08-0.19) | 0.1 | (0.07-0.19) | 0.13 | (0.09-0.22) | 0.12 | (0.08-0.18) | 0.13 | (0.08-0.23) | 0.12 | (0.1-0.14) | 0.18 | | (0.06-0.19) |  |
| **3+4** | **No** | **Months** | - |  | 13 | (11-16.25) | 27.5 | (24.25-33.75) | 40 | (36-48) | 51 | (45.5-62) | 64.5 | (61.5-67.25) | 83 |  | - | |  |  |
|  |  | **N of scans** | 65 |  | 62 |  | 46 |  | 31 |  | 19 |  | 4 |  | 1 |  | - | |  |  |
|  |  | **Lesion** | - |  | 13 | (21%) | 17 | (37%) | 13 | (41.9%) | 10 | (52.6%) | 2 | (50%) | 1 | (100%) | - | |  |  |
|  |  | **Progression** | - |  | 11 | (17.7%) | 16 | (34.8%) | 10 | (32.3%) | 11 | (57.9%) | 2 | (50%) | 0 |  | - | |  |  |
|  |  | **PSA** | 6.7 | (5-8.17) | 6 | (3.93-8) | 8.0 | (6.55-9.78) | 8.0 | (6-10) | 9.1 | (5.64-15) | 8.9 | (6.03-12.43) | 5.6 |  | - | |  |  |
|  |  | **Volume** | 46 | (31-66) | 47 | (35.75-69.75) | 49.5 | (40-69.25) | 64 | (51.25-77) | 66 | (38-101.5) | 81 | (62.75-94.25) | 75 |  | - | |  |  |
|  |  | **PSAD** | 0.12 | (0.09-0.21) | 0.10 | (0.07-0.16) | 0.13 | (0.08-0.19) | 0.13 | (0.09-0.2) | 0.15 | (0.06-0.17) | 0.13 | (0.06-0.23) | 0.07 |  | - | |  |  |
|  | **Yes** | **Months** | - |  | 12 | (9.0-16.5) | 25 | (22.75-30.25) | 39 | (34.5-48) | 55 | (45.75-67.5) | 74 | (68.25-85.75) | - |  | - | |  |  |
|  |  | **N of scans** | 83 |  | 66 |  | 47 |  | 32 |  | 16 |  | 6 |  | - |  | - | |  |  |
|  |  | **Lesion** | 83 |  | 54 | (81.8%) | 30 | (63.8%) | 24 | (75%) | 11 | (68.8%) | 4 | (66.7%) | - |  | - | |  |  |
|  |  | **Progression** | - |  | 19 | (28.8%) | 19 | (40.4%) | 16 | (50%) | 6 | (37.5%) | 2 | (33.3%) | - |  | - | |  |  |
|  |  | **PSA** | 7.23 | (4.9-9.58) | 9 | (5.68-11) | 7.75 | (5.53-10.75) | 7.95 | (4.2-10.34) | 7.16 | (6.2-7.2) | 9.22 | (7.61-10.61) | - |  | - | |  |  |
|  |  | **Volume** | 42 | (30-66) | 42 | (29-63.5) | 41 | (30-55) | 39 | (30-54.25) | 62 | (41.25-78) | 80 | (79-86.5) | - |  | - | |  |  |
|  |  | **PSAD** | 0.15 | (0.1-0.22) | 0.16 | (0.11-0.26) | 0.16 | (0.14-0.24) | 0.15 | (0.12-0.28) | 0.11 | (0.1-0.18) | 0.12 | (0.1-0.13) | - |  | - | |  |  |

| Supplementary Table 2B: Yearly MRI outcomes (first 8 years), stratified by Gleason grade and disease MRI visibility at baseline. | | | | | | | | | | | | | | | | | | |
| --- | --- | --- | --- | --- | --- | --- | --- | --- | --- | --- | --- | --- | --- | --- | --- | --- | --- | --- |
| Gleason | **Visible** | **Months** | **0-12** |  | **13-24** |  | **25-36** |  | **37-48** |  | **49-60** |  | **61-72** |  | **73-84** |  | **85-96** |  |
| 3+3 | **No** | **N of scans** | 119 |  | 173 |  | 146 |  | 118 |  | 116 |  | 70 |  | 53 |  | 41 |  |
|  |  | **Lesion** | 26 | (21.8%) | 36 | (20.8%) | 35 | (24.0%) | 27 | (22.9%) | 23 | (19.8%) | 17 | (22.1%) | 18 | (34%) | 14 | (34.2%) |
|  |  | **Progression** | 17 | (14.3%) | 32 | (18.5%) | 33 | (22.6%) | 27 | (22.9%) | 22 | (19%) | 13 | (17.7%) | 19 | (35.9%) | 16 | (39%) |
|  |  | **PSA** | 6.2 | (4.2-9.5) | 6 | (4.3-9) | 5.9 | (3.7-9) | 8.75 | (5.5-11.1) | 8.35 | (4.7-11.5) | 9.63 | (5.6-13.0) | 8.12 | (6.4-10) | 9.13 | (5.8-11.3) |
|  |  | **Volume** | 50 | (39.5-69.5) | 55 | (36-71) | 50.5 | (36-81) | 55 | (40-73) | 57.5 | (42-86.75) | 75.5 | (57-101.5) | 60 | (47-86) | 63 | (47.5-75.5) |
|  |  | **PSAD** | 0.12 | (0.07-0.16) | 0.12 | (0.08-0.17) | 0.11 | (0.07-0.17) | 0.15 | (0.1-0.22) | 0.11 | (0.09-0.16) | 0.11 | (0.07-0.18) | 0.15 | (0.11-0.18) | 0.13 | (0.11-0.17) |
|  | **Yes** | **N of scans** | 79 |  | 113 |  | 101 |  | 89 |  | 76 |  | 50 |  | 34 |  | 26 |  |
|  |  | **Lesion** | 52 | (65.8%) | 72 | (63.7%) | 57 | (57%) | 58 | (65.2%) | 43 | (56%) | 29 | (57.1%) | 19 | (55.9%) | 15 | (57.7%) |
|  |  | **Progression** | 12 | (15.2%) | 17 | (15%) | 24 | (24%) | 34 | (38.2%) | 27 | (34.7%) | 21 | (42.9%) | 13 | (38.2%) | 11 | (42.3%) |
|  |  | **PSA** | 7.0 | (5.3-9.9) | 6.5 | (5.1-8.7) | 7.3 | (4.7-10) | 7.49 | (5.6-10.3) | 7.9 | (5.8-10.5) | 8.6 | (5.4-13.9) | 10·5 | (8.4-12.9) | 6.65 | (5.4-8.7) |
|  |  | **Volume** | 55 | (32-68.5) | 48 | (37.5-65) | 60.5 | (41-77.25) | 56 | (44.5-70) | 68 | (50-88) | 65 | (50.5-73.5) | 65 | (53.5-81.5) | 72 | (51-88) |
|  |  | **PSAD** | 0.14 | (0.09-0.18) | 0.13 | (0.08-0.22) | 0.13 | (0.07-0.2) | 0.15 | (0.08-0.22) | 0.09 | (0.08-0.2) | 0.12 | (0.09-0.18) | 0.16 | (0.09-0.19) | 0.09 | (0.08-0.12) |
| 3+4 | **No** | **N of scans** | 30 |  | 38 |  | 37 |  | 27 |  | 14 |  | 8 |  | 2 |  | - |  |
|  |  | **Lesion** | 3 | (10%) | 8 | (21.1%) | 17 | (46.0%) | 14 | (51.9%) | 5 | (35.7%) | 4 | (50%) | 1 | (50%) | - |  |
|  |  | **Progression** | 4 | (13.3%) | 3 | (7.9%) | 16 | (43.2%) | 14 | (51.9%) | 5 | (35.7%) | 3 | (37.5%) | 0 |  | - |  |
|  |  | **PSA** | 6.4 | (3.8-7.8) | 6.3 | (4.3-9) | 8.5 | (7.4-10.4) | 6.4 | (5.3-9.1) | 9.2 | (5.1-11.4) | 7.45 | (6.5-11.2) | 7.85 | (6.7-9) | - |  |
|  |  | **Volume** | 46 | (35-59) | 49.5 | (37.75-69.75) | 54.5 | (34.5-71.25) | 51.5 | (40-75.75) | 55 | (47-72) | 100 | (89.75-109.5) | 57 | (48-66) | - |  |
|  |  | **PSAD** | 0.09 | (0.07-0.16) | 0.11 | (0.07-0.16) | 0.16 | (0.12-0.28) | 0.16 | (0.13-0.18) | 0.09 | (0.07-0.16) | 0.06 | (0.05-0.14) | 0.17 | (0.12-0.21) | - |  |
|  | **Yes** | **N of scans** | 41 |  | 39 |  | 36 |  | 24 |  | 15 |  | 6 |  | 3 |  | 2 |  |
|  |  | **Lesion** | 34 | (82.9%) | 29 | (74.4%) | 25 | (68.6%) | 18 | (73.9%) | 9 | (60%) | 5 | (83.3%) | 3 | (100%) | 1 | (50%) |
|  |  | **Progression** | 9 | (22%) | 15 | (38.5%) | 18 | (48.6%) | 10 | (39.1%) | 5 | (33.3%) | 4 | (66.7%) | 3 | (100%) | 0 |  |
|  |  | **PSA** | 9.0 | (4.6-11) | 7.5 | (5.7-8.9) | 9 | (6.8-12.6) | 7.3 | (5.7-10.1) | 7.85 | (3.7-9.8) | 6 | (5.6-11.5) | 8 |  | 12 |  |
|  |  | **Volume** | 38 | (27-51.25) | 43 | (31.5-55.5) | 43.5 | (30.5-64) | 45 | (30.5-71.75) | 51 | (32-60) | 54 | (29.25-106) |  |  | 93 |  |
|  |  | **PSAD** | 0.15 | (0.11-0.23) | 0.16 | (0.14-0.23) | 0.19 | (0.15-0.43) | 0.12 | (0.08-0.22) | 0.13 | (0.1-0.19) | 0.17 | (0.13-0.4) |  |  | 0.13 |  |

**Supplementary Table 2:** **Serial (A) and yearly (B; years 1-8) MRI outcomes of imaging-based AS.** Information including serial MRI number or time elapsed from the first mpMRI (in months) and the number of scans demonstrating a visible lesion or signs of radiological progression, along with PSA (ng/mL), prostate volume (mL) and PSA density (PSAD, ng/mL^2^) measurements are displayed. Absolute numbers or medians are shown, with percentages or IQRs (respectively) in brackets. There is a decrease of the total number of men undergoing a scan over time as more patients leave AS. Although the proportion of MRIs with a lesion remained relatively stable over time, there was a gradually increasing proportion of MRIs with signs of radiological progression. Median PSA and prostate volume also increased over time or with each additional MRI, but longitudinal changes in median PSA density were more erratic.
